# Laser-Fabricated Micro/Nanostructures: Mechanisms, Fabrication Techniques, and Applications

**DOI:** 10.3390/mi16050573

**Published:** 2025-05-13

**Authors:** Andrei Teodor Matei, Anita Ioana Visan, Irina Negut

**Affiliations:** 1 IT Center for Science and Technology, 25 No. Av. Radu Beller, 011702 Bucharest, Romania; matei.andrei5@gmail.com; 2 National Institute for Laser, Plasma and Radiation Physics, 409 Atomistilor Street, P.O. Box MG 36, 077125 Magurele, Romania

**Keywords:** laser processing, micro/nanostructures, optoelectronic devices, surface engineering, light enhancement, photovoltaics, photodetectors

## Abstract

The rapid evolution of optoelectronic devices necessitates innovative fabrication techniques to improve their performance and functionality. This review explores the advancements in laser processing as a versatile method for creating micro- and nanostructured surfaces, tailored to enhance the efficiency of optoelectronic applications. We begin by elucidating the fundamental mechanisms underlying laser interactions with materials, which facilitate the precise engineering of surface topographies. Following this, we systematically review various micro/nanostructures fabricated by laser techniques, such as laser ablation, laser-induced periodic surface structures (LIPSS), and two-photon polymerization, highlighting their unique properties and fabrication parameters. The review also delves into the significant applications of these laser-fabricated surfaces in optoelectronic devices, including photovoltaics, photodetectors, and sensors, emphasizing how tailored surface structures can lead to improved light absorption, enhanced charge carrier dynamics, and optimized device performance. By synthesizing current knowledge and identifying emerging trends, this work aims to inspire future research directions in the design and application of laser-fabricated micro/nanostructures within the field of optoelectronics. Our findings underscore the critical role of laser technology in advancing the capabilities of next-generation optoelectronic devices, aligning with the scope of emerging trends in device engineering.

## 1. Introduction

Micro/nanostructures represent artificial structures with special dimensions, levels and properties that are different from bulk materials. They have become essential in the past 20 years in several application fields [1].

Surface micro/nanostructures have become more and more popular in different engineering domains [2], including optical devices [3], the biomedical field [4], photovoltaics [5], electronics [6], and others. In the biomedical field, these structures can be used to create different types of biocompatible materials , such as biomimetic structures [6], to fabricate biosensors [7], and to create drug delivery systems, which have great potential in tissue engineering and diagnosis [8]. Biomimetic structures have different shapes for different types of applications. For example, micro/nanopatterns possess the ability to memorize and recover to specific structures and are used for cell capture/release [9] or anti-bioadhesion [10,11]. Also, shape of micro/nano-fibers is characterized by high porosity and permeability with a thinner layer; their usage is found in various domains, including tissue engineering [12], pollution [13] and photophysics [14]. A promising biomimetic structure is porous scaffolds, which are used for treating various defects, including traumas, congenital malformation, aging, or bone implants, by providing suitable requirements for tissue growth and cell proliferation [11]. Biosensors made by micro/nanostructures are analytical devices containing biological sensing elements to obtain the biological signal, a physicochemical transducer to convert the biological signal, a signal processor, and a detector [15,16]. Furthermore, nano-biosensors are biosensors from the fourth generation of nanotechnology and biotechnology development. These biosensors have an excellent ability to detect and analyze the presence of nanomaterials or molecules, and they have been applied to several fields, including clinical diagnosis, military science, environmental protection, and food analysis [15,16]. Biosensors can detect compounds such as carbohydrates, alcohols, vitamins, amines, amides or microorganisms [17]. On the other hand, surface micro/nanostructures can be used as conductive materials for drug delivery systems. The scientific literature offers many examples of materials used for surface micro/nano-drug delivery systems, and they could be classified into two groups: organic and inorganic materials [18]. One of the promising conductive inorganic materials is represented by a unique group of polymer nanocomposites, which possess the ability to conduct by incorporation of a polymer matrix with nanofillers [19]. Conductive polymers possess metal-like electrical properties while maintaining a polymer’s mechanical properties, making them suitable for biomedical applications due to enhanced responsiveness, reduced impedance and high charge transfer capacity [20]. Among other inorganic materials used as drug-delivery systems, such as casein, albumin or fibroin, one important material is gelatin because it is considered biocompatible and biodegradable.

Regarding optical devices, surface micro/nanostructures can be used to manufacture thin-film solar cells [21], microcavities [22], diffractive optical elements or surface plasma polarization (SPP) [23]. These devices pursue highly localized optical fields based on diffractive and refractive optics. Also, optical devices can detect small entities based on different principles of absorption, reflectance, emission, and fluorescence, utilizing light–matter interactions. Micro/nano-optical sensors (MNOs) are characterized by real-time and fast detection, which shows great potential in biological detection and analysis [24]. A sub-type of MNOs are the micro/nano-optical fibers (MNFs), which provide a unique way to explore fiber-optic technology. With their wavelength or sub-wavelength transverse dimensions, MNFs are ideal for nano-waveguides, exhibiting several properties, such as surface field enhancement, tight optimal confinement to manipulate light, and connecting fiber optics with near-field optics [25]. Notably, another interesting optical device that implies the usage of surface micro/nanostructures is a photodetector. A photodetector converts the optical signal into an electrical signal, having applications in different domains, including medicine, biology, optical communication or missile detection [26]. Moreover, using 1D metal-oxide nanostructures for building photodetectors provides excellent sensitivity, fast response speed and superior quantum efficiency due to their well-controlled morphology and composition. These characteristics make the photodetector suitable for several domains, especially UV irradiation detection [27]. Also, surface micro/nanostructures can be used to enhance the detection of molecules by Raman spectroscopy. Surface-enhanced Raman scattering (SERS) is a powerful analysis technique based on the localized surface plasmon resonance (LSPR) effect. Metal nanoparticles, especially noble nanoparticles, have extensive application in the preparation of SERS because of their strong LSPR and their affinity with the thiophenol molecule, which is widely used for Raman detection [28,29]. For example, surface micro/nanostructures used as SERS substrate is represented by gold and zinc oxide (Au/ZnO) composites by coating Au nanospheres over ZnO films to enhance the photocatalytic activity and to develop a stronger Raman signal [29].

To produce surface micro/nanostructures, there are several ways mentioned in the scientific literature, including top-down and bottom-up methods. Even though both approaches have their own advantages and disadvantages, bottom-up methods are mostly used to fabricate surface micro/nanostructures [30]. Some of these approaches include chemical vapor deposition (CVD) [31], electrochemical deposition [32], sol–gel coating [33], lithography [34], and magnetron sputtering [35]. However, the surface of materials fabricated by this method may face some challenges regarding technical procedures, limitations of these methods, and environmental pollution. For example, the chemical vapor deposition method enables control over structure and properties of the material and is developed to cover a large-area deposition with uniform coatings. It is suitable for fast deposition of coatings with applicability in different domains. Nevertheless, CVD presents some drawbacks. CVD is based on complex equipment or custom-built equipment, which includes a furnace [36], a reaction chamber [37], or different quartz tubes [38], which are difficult to maintain at functional parameters after a series of depositions. Also, CVD can be dependent on toxic precursors that can have a real environmental impact. The electrochemical deposition method could provide a fast and facile process because it does not require complex experimental conditions. Room conditions, such as pressure and temperature, are enough for electrochemical deposition. Moreover, this method is very accessible, electrochemical setup is often available in every laboratory and the experiments can be performed without having to follow complex protocols [39]. However, surface morphology is hard to control and is dependent on the electrolyte solution, the values of the parameters used, and the electrodes used. Also, electrochemical deposition is suitable for conductive micro/nano-materials, making it difficult to create surface micro/nano-materials that are non-conductive. Benefits and drawbacks can be determined for sol–gel coatings as well. The sol–gel approach for creating surface micro/nanostructures suggests a method that controls the size of the particles and the morphology by changing and monitoring the reaction parameters [40]. Considered as “soft-chemistry”, the sol–gel method is a low-cost process involving low temperatures and high yield [40,41]. The disadvantages of the sol-gel process include the possibility of phase separation during heat treatment, long preparation compared to other methods, and thickness limitations regarding thick films [42]. Lithography represents another method of fabricating surface micro/nanostructure, which is widely used and mentioned in scientific literature. This method is subdivided into different processes based on the energy source: interference lithography [43], X-ray lithography, electron beam lithography, colloidal lithography, atomic force microscope nanolithography, and many more. Every process has different benefits, drawbacks and applications. For example, the interference lithography allows fabrication of the 1D, 2D and especially 3D patterns using coherent beams of light. The 3D micro/nanostructures determined are large-area and defect-free, having a wide range of applications and being a fast and cheap process [44]. However, interference lithography requires a high operating environment and high equipment cost due to the number of optical components used and optical anti-vibration platforms [45]. The magnetron sputtering technique uses a solid target from which atoms are ejected and then deposited onto a substrate where thin films are grown. It provides unique properties and functionalities to these fabricated surface nano/microstructures that are hardly achieved using any other conventional method [46]. Nevertheless, the deposition rate of magnetron sputtering is slow, can provide a non-uniform morphological surface, and for this instance, this method is used with other techniques [47] and has high energy consumption [35].

Recent developments in the literature target laser deposition of surface micro/nanostructures that have been widely used for the last few decades [48]. As a synthetic and microfabrication technique, lasers have several advantages over conventional methods for fabricating surface micro/nanostructures, including fast processing and a cost-effective technique [49]. Surface micro/nanostructures fabricated by lasers have applications in different domains, including optoelectronics [50], surface plasma resonance (SPR) [51], biomedical [52], electronic devices [49], and other fields. Optoelectronic devices, which are usually made by semiconductor compounds, such as InP, GaAs, GaSb, indium tin oxide (ITO) or fluorine tin oxide (FTO), have generated high interest for usage as photodetectors, photodiodes, light-emitting diodes (LEDs) and liquid-crystal displays (LCDs) [53,54]. Their main function is to convert light energy into electric energy or vice versa [55]. Laser deposition for fabricating optoelectronics represents an efficient approach to creating nanomaterials with adjustable properties, allowing new features and designs for optoelectronic devices [53].

Relating to the environmental problem for laser-assisted techniques is not an issue because it enables direct synthesis of nanomaterials in different environments, being environmentally friendly with less energy consumption. Laser-assisted technologies can produce nanomaterials of smaller sizes and avoid the use of toxic compounds or unfriendly environmental reactants. These technologies can be divided into four main types of laser treatment: remelting techniques, remelting-free techniques, evaporation techniques, and techniques for the formation of thin and hard coatings [56]. Moreover, the laser technique prevents the necessity of masks and lithography, enhancing the production domain of devices and the flexibility in the geometric structure of micro/nanostructures [49]. Overall, laser-assisted technologies provide a quality approach to create surface micro/nanostructures with environmentally friendly processes, a fast procedure, and efficient control over the size of these surfaces.

In this review, we focus on different laser techniques that are used to fabricate various micro/nano-surface structures suitable for improving optoelectronic device performance, pointing out the mechanism of each laser technique, analyzing the different micro/nanostructure surfaces, and determining other applications of these structures.

## 2. Mechanism of Laser Processing Surfaces

**Figure 1:**
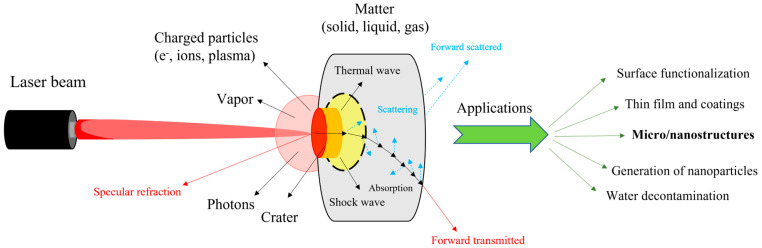
Interaction between the laser beam and matter along with several applications (adapted from [57]).

Laser techniques offer a valuable tool for the fabrication of micro/nanostructures, providing innovative properties for designing optoelectronic devices and enhancing their performance. The interaction of a laser beam with matter is a fundamental process where effects such as reflection, refraction, scattering, and absorption [57] guide light transport, producing different types of waves. This phenomenon leads to the emission of charged particles, photons, vapor or crates. Figure 1 presents a schematic version of this phenomenon along with several applications arising from this interaction.

Mathematical models and quantitative analyses of light–matter interactions are crucial for understanding the underlying physical processes in modern physics [58]. The quantum Rabi model (QRM) serves as a simple yet comprehensive framework describing interactions between a two-level system and a quantized light field [59]. This model highlights the complexities of these interactions, especially as the coupling strength approaches the natural frequencies of the involved systems, thereby entering the ultrastrong coupling regime [60].

The dynamics of light–matter interactions can be effectively studied using cavity quantum electrodynamics, which allows for the examination of single atoms coupling to single photons [61].

This setting facilitates the application of advanced theoretical approaches, such as the Jaynes–Cummings Hamiltonian, to elucidate the coherent and incoherent processes involved in radiative decay [62].

Continuous measurement and feedback mechanisms have been developed to manipulate many-body quantum states, revealing rich quantum correlations and opening possibilities for applications in quantum information processing [63].

Furthermore, quantum spectroscopic techniques leverage entangled light to enhance the sensitivity of measurements of light–matter interactions, demonstrating the multifaceted mechanisms at play [64].

Collectively, these mathematical models and their rigorous analyses provide essential insights into light–matter interactions, which span a wide array of applications, from fundamental physics to innovative technologies in quantum communication and beyond [65].

Moreover, the interaction between lasers and materials can be quantitatively described using the two-temperature model, which distinguishes between electron and lattice temperatures during ultrafast laser pulses. The model accounts for electron thermal properties, energy transfer to the lattice, and the laser’s energy deposition. For nanosecond lasers, thermal diffusion becomes the dominant mechanism, involving material properties, such as density, heat capacity, and thermal conductivity. These models explain key phenomena in laser–material interactions; thus, in the case of LIPSS formation, the results from interference between incident laser light and surface-scattered waves, with the period depending on the laser wavelength, refractive index, and angle of incidence; meanwhile, for ablation thresholds, the scale with pulse duration is a critical factor in precision machining applications [66].

Also, another mathematical model describes the laser-induced temperature evolution in a cylindrical solid material (like semiconductors) by counting two-photon absorption (TPA) processes. This equation simulates how thermal energy is deposited and propagates within the material over time and space when it is irradiated by a pulsed laser beam. This model is useful for the prediction of temperatures inside IR optical materials to minimize thermal damage during laser irradiation and to assess the impact of nonlinear absorption. This is described by the following partial differential equation [67]:

(1) $$\partial^{2}T/\partial r^{2}+\left(1/r\right)\partial T/\partial r+\partial^{2}T/\partial z^{2}-\left(1/\alpha \right)\partial T/\partial t=-A\left(r,z,t\right)/k$$

Another equation describes the material removal rate during laser micro-machining using the following formula and is important for optimizing the efficiency of cutting or drilling processes:

(2) $$MRR=\frac{w_{1}-w_{2}}{\rho *t}$$

where: w_1_—initial weight of the material;

w_2_—final weight of the material;

*ρ*—density of the material (g/mm^3^);

*t*—total machining time (s) [68].

Such quantitative frameworks bridge fundamental physics to practical fabrication outcomes.

**Figure 2:**
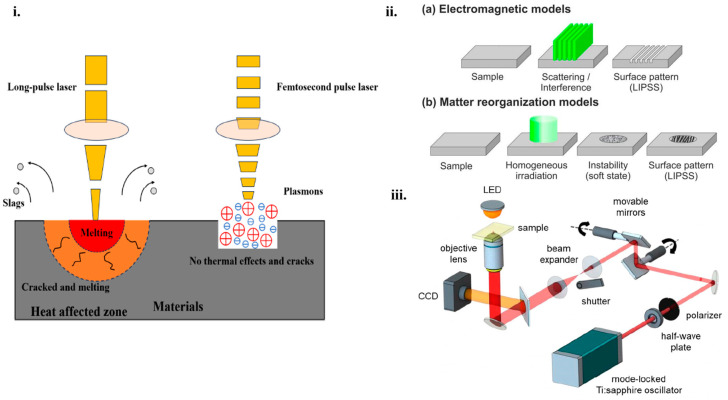
(**i**). Schematic representation of the interaction process between a long-pulsed-width laser and the femtosecond laser with the material [73]. (**ii**). (**a**) Electromagnetic model of static thermal melting, (**b**) matter reorganization models [75]. (**iii**). Representation of the femtosecond laser microfabrication via TPP setup, showing the basic components of the system [77].

Different types of lasers are used for processing the surface of micro/nanostructures, including pulsed laser ablation [69], laser-inducted periodic surface structures (LIPSS) [70] and two-photon polymerization (TPP) [71]. Depending on the pulse width, the pulsed laser consists of microsecond, picosecond and femtosecond pulses, finding their applications in different domains. For example, millisecond lasers have a long pulse duration and are suitable for cutting and drilling. However, exposing the target to this type of laser can cause thermal damage when used for surface texturing or structuring due to the high energy beam that could melt a portion of the surface because of the increasing heating during extended pulse radiation. Nanosecond lasers are the most common lasers used for fabricating micro/nanostructures on surfaces. It is usually used to remove excess material due to the necessary pulse time that provides the thermal waves propagation into the substrate. This type of laser is mainly used for rapid prototyping and micro-structuring. Regarding picosecond lasers, they provide low pulse energy but ultrahigh peak power. Compared to the nanosecond and microsecond lasers, they possess less thermal damage, better precision and faster processing. Picosecond lasers are commonly used for precise processing, medicine, or micro/nanostructuring. Femtosecond lasers have an extremely short pulse width and have the capacity for creating an ultra-strong electric field with extremely high power [72]. The femtosecond laser source is the most used for processing micro/nanostructures because it provides ultrafast pulses where all the energy is focused on a small area and it can adjust material properties [2,69,70]. In comparison with long-pulse-width, the femtosecond laser provides significant advantages related to the interaction between the laser source and material, such as an accurate ablation threshold, wide applicability, and the avoidance of thermal effects into the material [73]. This interaction starts when photons are absorbed by electrons, and the thermalization of hot electrons creates a high electron temperature. After a few picoseconds in the interaction between the laser and material, a thermal effect is considered to be active and, as a result, this interaction is very important for micro/nanostructuring [74]. Figure 2i presents the comparison between the interaction process of a long-pulsed-width laser and femtosecond laser with the material [73]. LIPSS are a phenomenon that occurs on any type of material, especially on solids after irradiation with linearly polarized radiation. LIPSS has received much attention, especially using femtosecond laser pulses, for having various applications in fields such as optical information storage, optoelectronics, or optical waveguides. In order to research and study the mechanism of the femtosecond laser on LIPSS, a classification of LIPSS has been proposed according to their ratio of spatial periods to the irradiation wavelength and the polarization direction of the laser beam. Also, the formation of LIPSS can be divided into two classes, electromagnetic theories and matter reorganization theory. The electromagnetic theories describe the interaction of electrons and magnetic radiation with microscopic surfaces. Meanwhile, the matter reorganization theory describes the way the surface matter is redistributed. Both theories are schematically represented in Figure 2ii where (a) presents a static thermal melting from a sample and (b) presents a dynamic response due to self-organization.

Using the TPP technique, it is possible to fabricate 3D microstructures with arbitrary geometries, with applications in numerous domains, such as photonics, micromechanical systems, or bioscience [75]. It presents several advantages over conventional techniques involving direct fabrication of complex micro/nanostructures, avoiding a time-consuming fabrication process. This technique can enhance the performance of optoelectronic devices by optimizing the fabrication parameters of micro/nanostructures [76]. The basic components of a system with a TPP femtosecond laser are presented in Figure 2iii. The laser beam is generated from a mode-locked Ti:sapphire oscillator, and then the pulse energy is adjusted using a half-wave plate and a polarizer. Two movable mirrors are used to scan the laser transversally and the objective lens is used for controlling the size and the thickness of the fabricated structures. To regulate the equipment parts, such as the shutter, movable mirrors, exposure time, and laser speed, control programs are set to be used. By combining movable mirrors with the 3D translational stage, microstructures are fabricated over a large area. During the process, the refractive index of the polymerized structure undergoes slight changes. When the TPP fabrication technique is completed, the sample is submerged in a suitable solvent to eliminate the unpolymerized material [77].

## 3. Micro/Nanostructures Fabricated by Laser Techniques

The advancements of laser-based methods offer significant potential for developing novel materials with enhanced functionalities across multiple scientific and industrial domains. Table 1 summarizes the laser techniques used for micro/nanostructure fabrication, highlighting their advantages, applications, and real-world research examples.

The performance improvements in optoelectronic devices achieved through laser-fabricated micro/nanostructures can be attributed to several key mechanisms. These advancements are primarily attributed to the creation of laser-induced periodic surface structures, two-photon polymerization, and laser ablation techniques. For instance, laser-induced periodic surface structures enhance light absorption and scattering, which is critical for photovoltaics and photodetectors [82]. This occurs due to the creation of subwavelength gratings that reduce reflection losses and increase the effective optical path length within the material [83]. Similarly, two-photon polymerization enables precise 3D structuring at the nanoscale, allowing for tailored photonic bandgaps and improved light confinement in waveguides or resonators. Laser ablation, on the other hand, facilitates the formation of defect-free surfaces and controlled doping profiles, which are essential for optimizing charge carrier dynamics in semiconductor devices [84]. These mechanisms collectively contribute to enhanced device efficiency, sensitivity, and functionality [85].

A comparative analysis of laser techniques (Table 2) reveals trade-offs in resolution, throughput, and material compatibility.

Notably, LIPSS excels in large-area patterning but struggles with non-metals, while TPP offers unmatched 3D resolution at the cost of speed. Hybrid approaches (e.g., laser-chemical etching) now achieve <10 nm features with throughputs of 1 cm^2^/min, addressing the ‘resolution-speed’ dilemma in industrial adoption [85].

The Laser-Induced Forward Transfer (LIFT) technique transfers material by focusing a laser pulse through a transparent support onto a thin source film, inducing its vaporization. The ejected material then deposits onto a nearby receiving substrate, typically separated by a few micrometers. Research indicates that for fragile substances, such as polymers and biological compounds, direct contact between the source and receiving surfaces yields optimal transfer fidelity and edge definition of the deposited features [89]. LIFT can be implemented using various laser sources, including UV excimer, Nd:YAG, argon-ion, and femtosecond lasers. Given the susceptibility of many transfer materials to atmospheric conditions, a vacuum or inert atmosphere is often necessary [90]. Initially employed for patterned metal film deposition, LIFT has broadened its scope to encompass oxides, biological materials [91], and intricate multilayer systems, such as polymer-based light emitters [92] and organic thin-film transistors [93]. For materials that are either transparent to the laser or prone to laser-induced damage, an energy-absorbing intermediate layer, known as a dynamic release layer, can be utilized to facilitate material ejection from the carrier substrate [94]. Furthermore, the temporal characteristics of ultrafast laser pulses play a crucial role in the LIFT process and the resulting feature size, a phenomenon linked to rapid electron and lattice dynamics. Studies have demonstrated that femtosecond pulses with short inter-pulse delays (below 500 fs) significantly influence the size of the deposited pixel, whereas the deposition area remains relatively constant for longer delays up to 10 picoseconds [95]. An alternative approach, parallel LIFT, utilizes arrays of microspheres as microlenses to focus an unfocused laser beam, enabling simultaneous transfer. In this method [96], polystyrene beads on a transparent substrate concentrate the light onto the source material, allowing for the creation of micron and submicron features on the receiving substrate [97].

LIPSS enhance light absorption and scattering, crucial for photovoltaics and photodetectors. They create subwavelength gratings that minimize reflection losses and extend the effective optical path length within materials [98]. This results in improved internal quantum efficiency in devices such as light-emitting diodes and laser diodes.

Two-photon polymerization enables the precise three-dimensional structuring of materials at the micro- and nanoscale, allowing for the creation of complex geometries with resolutions often below the diffraction limit [99]. This capability is crucial for fabricating photonic crystals with tailored bandgaps that control the flow of light at specific wavelengths [100], leading to the production of multifarious photonic devices, particularly the photonic crystals, which are promising candidates for integrated optical devices. Furthermore, TPP’s precision allows for the fabrication of waveguides and resonators with optimized geometries for enhanced light confinement, resulting in improved performance in optical applications [101].

Laser ablation is a technique that facilitates the formation of surfaces with reduced defects and enables controlled doping profiles in semiconductor materials. These characteristics are crucial for optimizing the movement of charge carriers within semiconductor devices, ultimately leading to enhanced device sensitivity and overall functionality [84].

### 3.1. LIPSS

**Figure 3:**
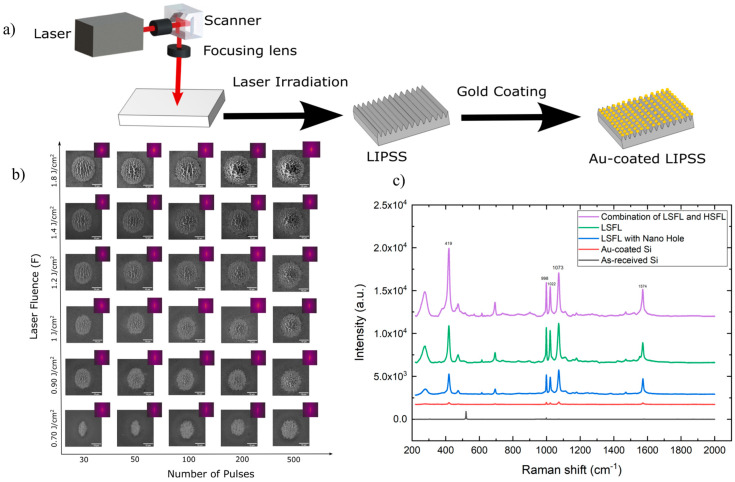
(**a**) Schematic of the experimental process followed for the fabrication of the gold nanoparticles on a silicon substrate, (**b**) SEM images with 2D FFT inset at the top right corner, illustrating LIPSS evolution on silicon under varied fluences and number of pulses, (**c**) the SERS spectra of thiophenol molecules in ethanol adsorbed on the as-received Si surface, Au-coated as-received Si surface, Au-coated combination of LSFL and HSFL surface, Au-coated LSFL surface, and Au-coated LSFL with nanohole surface [104].

The LIPSS technique has been highlighted since its discovery in 1965 for developing various devices. The key factors regarding this technology are related to the type of laser used. Critical factors, such as fluence, wavelength, pulse number, material properties, or polarization, can determine the size, shape, and orientation of the micro/nanostructures [102]. LIPSS presents a series of advantages compared to conventional techniques that include low sensitivity to processing materials, maintaining the surface intact due to the fact that they are subwavelength nanostructures and are beneficial in creating periodic subwavelength structures [67], high stability, flexibility, and processing efficiency [103]. To enhance the regularity of LIPSS, processing experiments have been classified into three categories: (i) sample pre-treatment, (ii) processing condition control, and (iii) sample post-treatment. For example, artificial seeds and thin film coatings are employed to control laser surface plasmon polarization interference, homogenize thermal distribution and enhance laser-surface periodic polarization (SPP) [103]. H. Vaghasiya and P. T. Miclea developed the formation of LIPSS in specific silicon substrates combining three different stages: integrated low-spatial-frequency LIPPS (LSFL) and high-spatial-frequency LIPSS (HSFL), LSFL and LSFL at irradiated spots to determine the impact on applications based on surface-enhanced Raman spectroscopy (SERS) [104]. To fabricate these silicon substrates a femtosecond Yb:KGW laser has been used with a pulse duration of 180 fs and an average power of 6 W. In order to achieve the amplification of the Raman signal, a gold thin film has been deposited onto these laser-fabricated silicon nanostructures, and the LIPSS experimental procedure is represented in Figure 3a. A valuable overview of the relationship between the parameters and the number of pulses can be determined by varying these parameters and visualizing the evolution of LIPSS in silicon. This dynamic evolution is represented in detail in Figure 3b. It is well observed that, at a value of approximately 0.70 J/cm^2^, LIPPS evolved from an elliptical circumference to circular shapes due to the increasing number of pulses and the changes that occur in the electric field. Increasing both of the parameters leads to the formation of nanohole arrays where localized ablation is visible. Figure 3c represents the SERS spectra of thiophenol molecules in ethanol absorbed on several surfaces. According to the Raman shifts and the represented SERS spectra at specific bands, the Raman enhancement factor efficiency follows the order of the three combined stages. In this way, the researchers proved the enhancement of SERS-sensing platforms through three distinct stages of LIPSS using a femtosecond laser.

**Figure 4:**
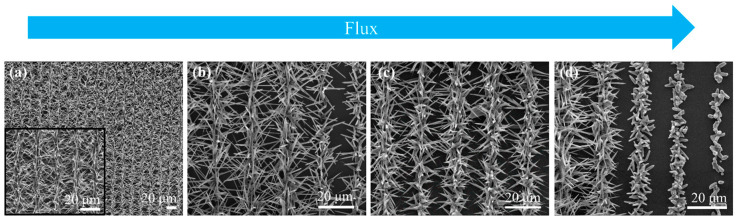
SEM images showing the evolution of the ZnO structures’ shape, grown on the a-c LIPSS substrate, along the direction of the Ar flux (blue arrow). On the left, (**a**) the obtained structures closer to the source and gas inlet; in the middle, (**b**,**c**) the structures obtained in the intermediate regions; and on the right, (**d**) the structures appear far away from the source [105].

B. Sotillo et al. demonstrated the use of LIPSS as templates for fabricating photocatalytic micro/nanostructures of ZnO [105]. ZnO, having a theoretical band gap of 3.3 eV, can provide different micro/nanaostructures with various morphologies and optically unique and present photocatalytic properties [106]. The ZnO structures were fabricated using a femtosecond laser with a Yb-doped fiber source, operating at a wavelength of 1030 nm and a pulse duration of 340 fs, and were subsequently deposited onto a silicon (Si) substrate. Following deposition, these structures were subjected to further growth in a horizontal tubular furnace at 900 °C under an argon (Ar) flux. SEM imaging confirmed the formation of ZnO structures on the Si substrate, revealing a significant correlation between the material deposition and the substrate’s position relative to the source. The highest concentration of ZnO nanostructures was observed in areas closest to the source (Figure 4a). Moving the source further away, the ZnO nanostructures become more elongated, and the density of the structures decreases (Figure 4b). Slight changes are observed moving from the source even further; the structures tend to become rod-like in shape (Figure 4c). With enough space between the source and the substrate (Figure 4d), the nanostructures are rod-shaped, with reduced length, until only the nucleation clusters become visible.

### 3.2. Two-Photon Polymerization (TPP)

TPP is a photochemical process initiated by a femtosecond laser guided tightly into the volume of photosensitive resins using a high-numerical-aperture objective (NA), and has gained prominence in materials chemistry, with numerous reviews available on the photophysics of this technique [89,107]. Initially introduced by Pao in 1965 for the polymerization of styrene via a two-photon process, two-photon polymerization (TPP) was later recognized as a key technique in direct laser writing (DLW) [89]. The resolution is a critical parameter in the fabrication of micro/nanostructures using two-photon polymerization (TPP). The fundamental structural units in TPP are represented by voxels or lines, and fabrication typically employs one of two scanning modes: pinpoint scanning or continuous scanning. The resolution in these modes is directly influenced by the size of the voxels, including their lateral and axial dimensions, as well as the width of the lines. Any inconsistencies in voxel formation can impact the overall accuracy of the fabricated structures. TPP offers significant advantages in the creation of highly precise 3D micro/nanostructures, making it a valuable technique for applications in optics, electronics, biomedicine, and communication technologies [107].

**Figure 5:**
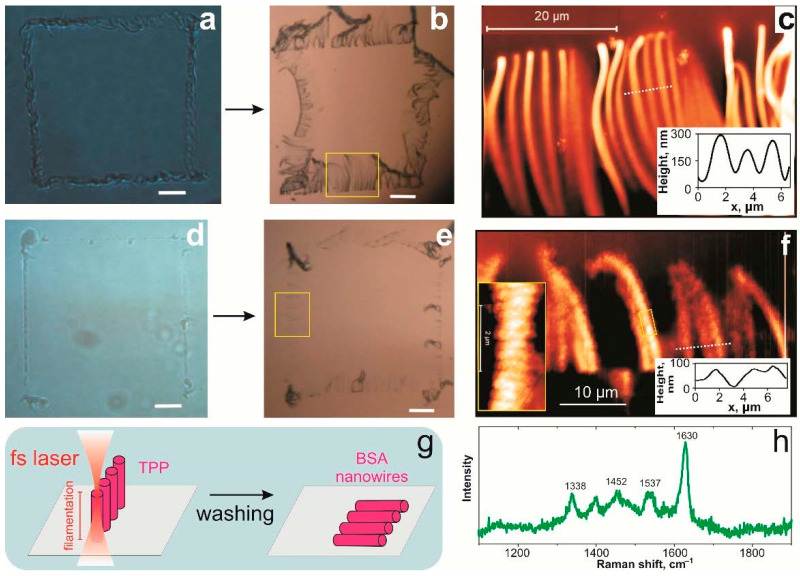
TPP of vertical BSA hydrogel nanowires: (**a**) Optical image of BSA hydrogel “walls” after polymerization under laser focused above the substrate; (**b**) optical image of “walls” after washing out non-polymerized BSA solution; (**c**) AFM image of “walls” marked by rectangular at (**b**). (**d**–**f**) the characterization of “walls” produced by laser focused close to substrate surface: optical image before (**d**) and after (**e**) washing; (**f**) AFM image of “walls” denoted by a rectangle of (**e**); insert images on right (**c**,**f**): cross-section of individual nanowires; left bottom insert of (**f**): enlarged AFM image of an individual nanowire with visible periodic sub-wavelength nanostructures; (**g**) scheme of BSA hydrogel nanowires fabrication by femtosecond pulsed laser; (**h**) Raman spectra of BSA hydrogel nanowires [108].

N. Nekrasov et al. proposed a method for fabricating a 3D-printed hydrogel from bovine serum albumin (BSA) with graphene oxide using the TPP approach via a femtosecond laser. The aim of their study is to synthesize albumin hydrogel nanowires and enhance them with graphene oxide. Using a tunable Ti:Sapphire femtosecond laser with a wavelength set to 715 nm with 140 fs, N.Nekrasov et al. obtained nanowires with good isolating properties and enhanced mechanical properties [108]. They initially observed that the diameter of the fabricated structures varied depending on the laser’s focus position. When the laser was positioned above the substrate, the 3D polymerized “walls” grew perpendicular to the surface (Figure 5a). After washing the sample, these “walls” broke apart, with their fragments falling onto the substrate (Figure 5b). The nanowires forming these “walls” measured approximately 30 μm in length and 320 nm in diameter, demonstrating a promising approach for achieving stable nanowires (Figure 5c,d). When the focus position was adjusted closer to the laser, the dimensions of the BSA hydrogel structures decreased to 20 μm in length and 70 nm in diameter, indicating a strong correlation between the nanowire size and the laser focus position. Additionally, sub-wavelength nanostructures with a periodicity of 300 nm were observed along the nanowire side “walls” [108].

**Figure 6:**
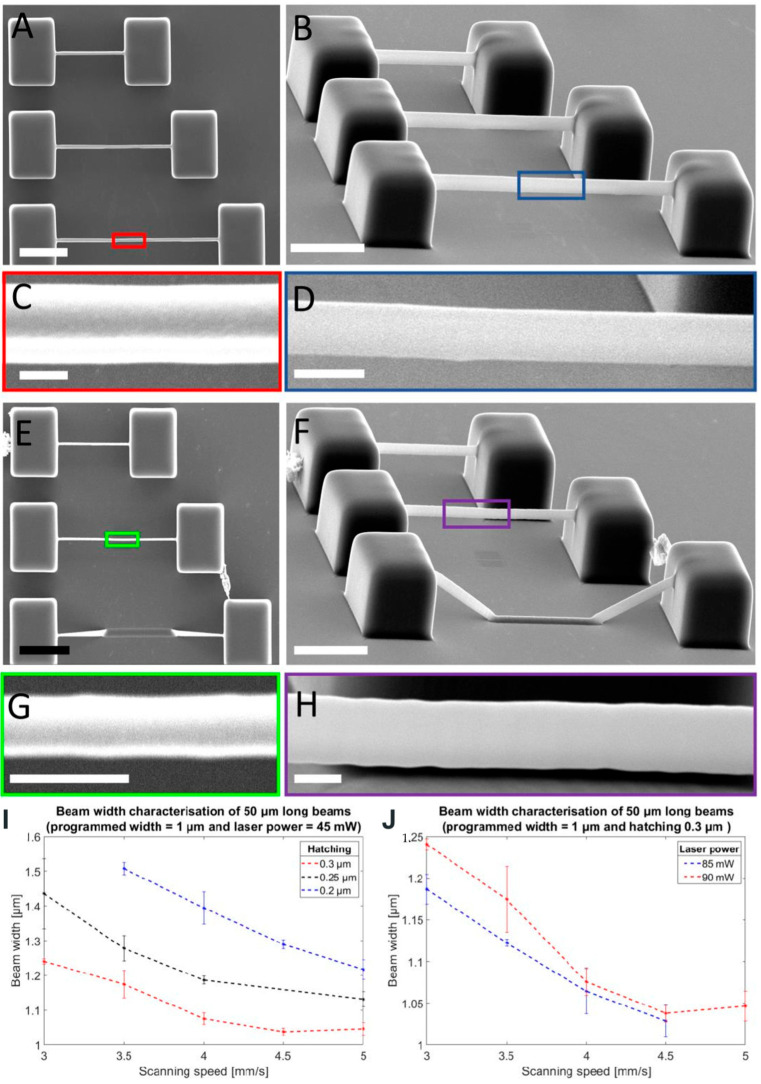
Representative SEM micrographs of 2PP-printed IP-PDMS beams. (**A**) Top view of beams with nominal lengths of 30, 50, and 70 μm and a nominal width of 1 μm. (**B**) Tilt view of 65 degrees of the beams in (**A**) (scale bar = 20 μm). (**C**) Top view close-up of the beam with a length of 70 μm. The beams printed with these settings have an average width of 1.46 ± 0.11 μm (*n* = 3; scale bar = 1 μm). Sixty-five-degree tilt view of the side of the printed beam indicated in (**B**). (**D**) Sixty-five-degree tilt view of the side of the printed beam indicated in (**B**). The measured thickness of the beam is 4.49 ± 0.05 µm (n = 3; scale bar = 5 µm). (**E**) Top view beams with nominal lengths of 30, 50, and 70 μm and a nominal width of 1 μm. (**F**) Tilt view of 65 degrees of the same beams as in (**E**). (**G**) Top view close-up of the beam with a length of 50 μm. (**H**) Sixty-five-degree angle view of the side of printed beam indicated in (**F**). Plots of the measured widths of 50 m long beams—(**I**) Graph showing the effect of both the scanning speed and the hatching distance on the resulting beam widths (constant laser power at 45 mW). (**J**) Effect of the laser power on the widths of the beams at different scanning speeds (constant hatching distance of 0.3 m) [109].

Another example of successfully fabricated microstructure by TPP has been studied by P. Van Anelta. and A. Accardo [109]. They investigated microscale 3D structures of elastomeric Inverse-Processed Polydimethylsiloxane (IP-PDMS) featuring tunable mechanical properties for cell biology applications, such as tissue engineering or in vitro disease models. Their study involved a comparative mechanical and morphological analysis of IP-PDMS. A femtosecond laser source with a wavelength of 780 nm, a maximum laser power of 50 mW, a repetition rate of 80 MHz, and a pulse length of 100 fs has been used. Important printing parameters, such as slicing distance, hatching distance or scan speed, have been investigated for obtaining optimal IP-PDMS beams with the required mechanical properties. To determine microstructures for cell biology experiments and applications, it is critical to mimic the features and structures of the in vivo cellular environment. This allows the advancement of the interaction between the biomaterial and the cell. They observed that very thin and narrow beams with long overhangs could be successfully printed using IP-PDMS, forming arrays of double-clamped beams with a designed width ranging from 1 to 4 μm (Figure 6A–H). In Figure 6I,J, two key printing parameters—hatching distance and laser power—were analyzed. A larger hatching distance and higher scanning speed resulted in the formation of narrower beams. Lower laser power further reduced the beam width compared to higher laser power settings. The fabrication strategy involved initially creating two supporting pedestals, from which each beam was printed sequentially, starting from the first pedestal and extending until it was connected to the second. The maximum reproducible beam length achieved was 70 μm. This approach enabled the formation of 3D freestanding microstructures with dimensions comparable to mammalian cells. By tuning the Young’s modulus of IP-PDMS to be two to three orders of magnitude lower than that of commercial 2PP acrylate polymers, the material demonstrated improved suitability for biomedical applications [109].

### 3.3. Laser Ablation

Laser ablation is an alternative approach for generating micro- and nanostructures directly from bulk materials. It has been recognized as a very powerful tool since 1960 when the first functional laser was built [110,111]. The peak power of lasers has significantly increased with the transition from continuous-wave lasers to pulsed lasers. This shift has expanded the range of applications, particularly in electrochemical energy storage and conversion, where advancements have continuously evolved over the years. Pulsed laser technology facilitated the deposition of active electrode materials in the 1990s, followed by pulsed laser printing of electrodes in the 2000s, ultimately leading to its successful industrial adoption for high-energy battery production in the 2010s. The high-power density concentrated near the focal point of pulsed lasers has made them highly effective for processing nearly all types of materials. This technique enables the ejection of macroscopic amounts of material from a solid surface, typically under vacuum conditions, or in gas and liquid environments, provided the medium does not attenuate the laser energy. In non-vacuum settings, laser ablation offers distinct advantages by promoting reactions within a dense, short-lived environment. Additionally, to ensure the conservation of energy and momentum, bimolecular bonding in the gas phase requires the presence of a third body. Compared to conventional fabrication techniques, laser ablation stands out due to its exceptional precision, high efficiency, cost effectiveness, and significant flexibility in material processing [110,111].

**Figure 7:**
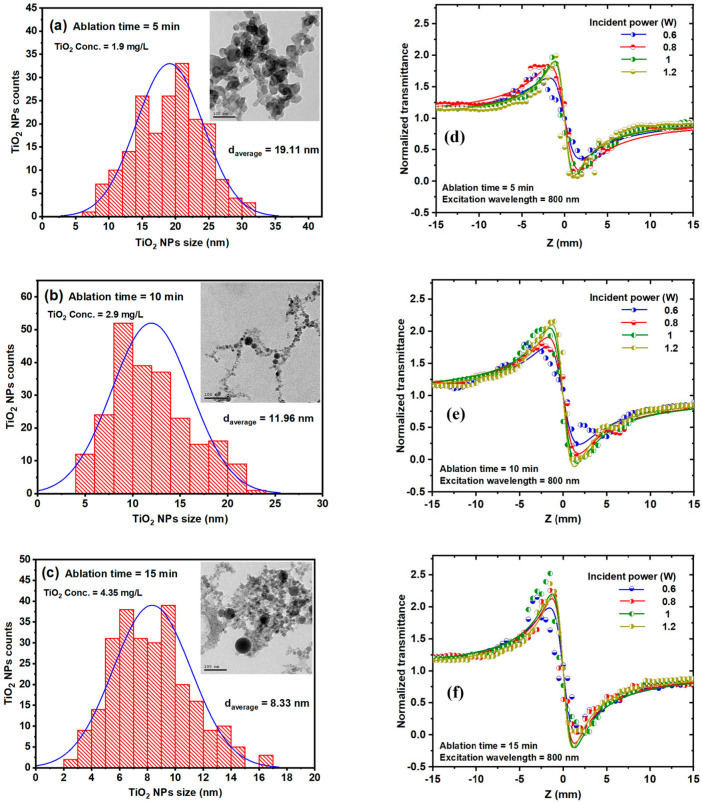
(**a**–**c**) depict the size distributions of TiO_2_ NPs colloids that were synthesized using various ablation times of 5 min, 10 min, and 15 min, respectively, (**d**–**f**) closed aperture Z-scan transmission of TiO_2_ NP colloids at different excitation wavelengths and ablation times [112].

F.A. Samad et al. [112] conducted an experimental study to characterize the nonlinear optical properties of titanium dioxide nanoparticles (TiO_2_) using femtosecond laser ablation along with the Z-scan approach. They synthesized TiO_2_ nanoparticle colloids using a harmonic Nd:YAG laser ablation system in distilled water. This technique, known as pulsed laser ablation in liquid (PLAL), offers several advantages over traditional chemical methods, including the production of stable metal colloids, the elimination of toxic chemicals, and precise control over nanoparticle size by adjusting key laser parameters, such as repetition rate, pulse duration, wavelength, and pulse energy. Using a harmonic Nd:YAG laser system with a pulse duration of 10 ns, a repetition rate of 10 Hz, and a wavelength of 532 nm, they successfully fabricated TiO_2_ nanoparticle colloids. The laser system operated with a maximum pulse energy of 1500 mJ per pulse, while the laser beam was focused through a convex lens, delivering an average power of 150 mW and 15 mJ per pulse. The titanium sample was immersed in 10 mL of distilled water within a rotating beaker at 177 RPM, ensuring uniform nanoparticle distribution and stabilization throughout the process. Thus, TiO_2_ nanoparticles were synthesized at different ablation times of 5, 10, and 15 min, respectively. F.A. Samad et al. initially determined the influence of ablation time on size, distribution and average size of TiO_2_ nanoparticles using high-resolution transmission electron microscopy. Varying the ablation time of 5, 10, and 15 min, the TiO_2_ NP colloids correspond to different concentrations of 1.9 mg/L, 2.9 mg/L, and 4.35 mg/L, respectively. Therefore, the average size of the TiO_2_ NP has been determined to be 19.11 nm for 5, 11.96 nm for 10, and 8.33 nm for 15 min of ablation, suggesting that increasing the ablation time will decrease the size of the NP. This phenomenon is suggested by the photo-fragmentation process, which becomes more influential and efficient with increasing ablation time. Figure 7a–c highlights the size distribution histograms and TEM images of TiO_2_ colloids for 5, 10, and 15 min ablations, corresponding to different concentrations discussed above. Optical properties of TiO_2_ colloids have been investigated by analyzing the effect of excitation wavelength on the nonlinear refractive index. The relation of various excitation wavelengths ranging from 750 to 850 nm and different ablation times with an incident power of 1 W varies as a function of the excitation wavelength for ablation times. At 5 min of ablation time, the nonlinear refractive index (NLR) exhibits a slight decrease with the increase of excitation wavelength. Contrary to this, at 10 min of ablation time, the NLR presents a slight increase as the excitation wavelength increases and this increase is more visible at 15 min of ablation time. The dependence of NLR index on the excitation wavelength at different ablation times is represented in Figure 7d–f. Several characteristics and factors, such as the thermal contribution and the band structure of the TiO_2_ NPs, influence this dependence of the NLR index on the excitation wavelength.

**Figure 8:**
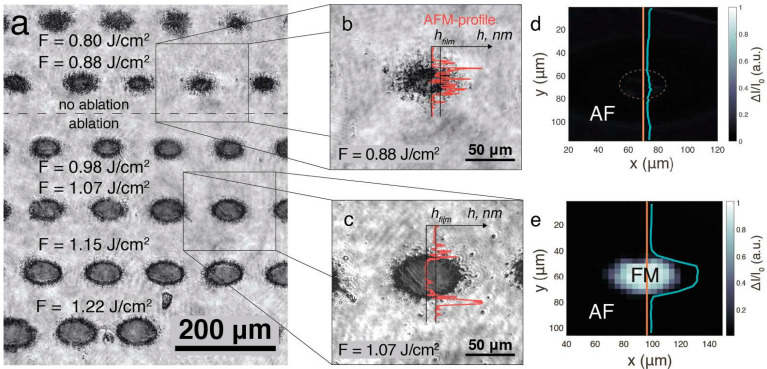
The microscopic pictures of the fs-laser produced structures in 45 nm thick B2-FeRh film: (**a**) Optical microscopy of the obtained film structures under F in a range from 0.8 to 1.25 J/cm^2^. Optical microscopy of the structure obtained by the pulse with (**b**) F = 0.88 J/cm^2^ and (**c**) F = 1.07 J/cm^2^. The red curve corresponds to the topographic profile measured with AFM. Film corresponds to the thickness of the film (45 nm). (**d**) S-MOKE pictures for the structures produced at the (**d**) F = 0.88 J/cm^2^ and (**e**) F = 1.07 J/cm^2^. The turquoise curve describes the spatial distribution of the MOKE signals obtained in the center along the orange line. AF stands for antiferromagnetic, FM stands for ferromagnetic [113].

In another experimental study made by P. Varlamov et al. [113], a femtosecond laser ablation was used to produce B2-ordered FeRh thin films in order to investigate their magnetic and morphological properties. They employed Scanning Magneto-Optical Kerr Effect (S-MOKE) microscopy to examine the magnetic phase after laser processing offering high spatial resolution and sensitivity. To prepare B2-ordered FeRh, they sputter deposited 45 nm thick films of equiatomic FeRh on an annealed MgO substrate at 600 °C. The annealing of the substrate proved to be crucial for achieving the B2-ordered structure. Laser processing was conducted exclusively under ambient conditions, ensuring a controlled fabrication environment. The process utilized uniform and precisely controlled femtosecond laser pulses, which were focused through a quartz lens under the following parameters: 800 nm wavelength, 50 fs pulse duration, 500 Hz repetition rate, and pulse energy up to 300 μJ. To create uniform femtosecond-laser-induced structures, the sample was systematically moved at a constant speed of 10 cm/s, ensuring consistency in pattern formation. To investigate the magnetic properties of FeRh structures formed after laser ablation, microphotographs of these structures reveal their morphology and dimensions and are represented in Figure 8a–c. Figure 8a represents the general optical microscopy of the obtained film structures under a fluence F in a range between 0.8 and 1.25 J/cm^2^. Figure 8b highlights a micrograph of a closer and detailed view of a structure fabricated by laser at a fluence of F = 0.88 J/cm^2^. At first glance, a dark structure is observed, measuring between 20 and 30 μm in diameter and surrounded by residual fragments. A red curve is visible in Figure 8b,c, representing the Atomic Force Microscopy (AFM) measurement of the relief profile. This analysis provides a detailed view of the morphology of the fabricated structures. In Figure 8c, compared to Figure 8a,b, the structure exhibits sharper and more defined boundaries, along with the formation of a 45 nm deep crater, accompanied by residual fragments at the base of the film. The Scanning Magneto-Optical Kerr Effect (S-MOKE) map in Figure 8d, corresponding to the position in Figure 8b, reveals an absence of detectable magnetic signals, indicating that the structure retained its antiferromagnetic state after laser irradiation. Conversely, Figure 8e presents a structure where material removal occurred, and magnetic signals were detected within its boundaries, suggesting a phase transition. These findings demonstrate a strong correlation between laser fluence and the magnetic behavior of FeRh thin films, confirming that laser ablation induces irreversible phase transformations and alterations in their magnetic properties.

## 4. Laser-Fabricated Micro/Nanostructures in Optoelectronic Devices

Optoelectronic devices are typically made using III-IV semiconductor compounds such as GaAs, InP, GaN and GaSb due to their direct band gap, and are different from electronic devices, which are silicon-based [54]. Whether composed of organic or inorganic materials, optoelectronic devices function as optical and electronic systems that emit, guide, modulate, and detect light, playing a crucial role in various technological applications [114]. These devices are used for a wide range of applications, such as communication, energy field, and health care, including light-emitting diodes (LEDs), amplifiers, optical modulators, solar cells, photodetectors, photovoltaics, and sensors. A key advantage of optoelectronic devices, particularly semiconductor-based ones, is their compact size, which allows for the fabrication of thousands of devices within a small area. Additionally, by modifying the composition of the multiple layers that make up their structure, these devices can be precisely tailored to suit a wide range of applications [54].

The history of optoelectronic devices can be dated to the early 1960 with the development of the LED and the semiconductor laser. In the late 1970s, molecular-beam epitaxy (MBE) and vapor-phase epitaxy (VPE) were further developed, which enabled thin layers to be grown reproducibly and to be achievable. This introduced the regime where quantum-confinement effects could be harnessed. The quantum well structures offered significant enhancements in laser performances, including narrower line width, extended wavelength tunability for material composition and carrier confinement. In the early 1980s, distributed feedback (DFB) lasers were developed, which favored long-distance communications, which opened the optimization and fabrication of optoelectronic devices, such as monolithic tunable lasers, optical modulators, and advanced photodetectors. In 2000, after about 20 years, Alferov and Kroemer became co-recipients of the Nobel Prize for physics for their work on the development of semiconductor heterostructures. This represents one of the most key discoveries and developments of optoelectronics, the full-range of developments of optoelectronic devices being more extensive [54].

Micro/nanostructures introduced inside or outside optoelectronic devices can significantly enhance their performance. These structures can improve the light absorption capacity and the efficiency of the surface plasmon polariton of optoelectronic devices. Laser fabrication technologies of micro/nanostructures for optoelectronic devices can lead to the fabrication of broadband, transparent anti-reflection surfaces with high efficiency, high precision and a low thermal effect. Thus, laser fabrication becomes a key part of optoelectronics devices due to the specific and unique optical and electronic properties that can be achieved by this technique [50]. In this review, we summarized some of the applications of optoelectronic devices, such as photodetectors, photovoltaic cells, sensors, and LEDs, fabricated by several laser techniques, including LIPSS, TPP, and laser ablation, to highlight the fabrication process, morphologies, and properties of micro/nanostructures for enhancement of optoelectronic device performance.

### 4.1. Photodetectors

Photodetectors propose a technology that converts an incident light signal to an electrical signal, which has been explored into many applications, such as temperature monitoring, thermal image technology and light communication systems.

Laser-fabricated micro/nanostructures significantly enhance photodetector performance by optimizing light–matter interactions. For instance, plasmonic nanostructures, often created via laser ablation, amplify localized electric fields, thereby boosting sensitivity in the UV-Vis spectrum [115] and enabling hot electron generation to extend the detection bandwidth [116], with demonstrated improvements in the responsivity of Si photodetectors up to 50 mA/W at specific wavelengths [116]. Furthermore, laser-induced periodic surface structures on semiconductors minimize carrier recombination losses, as evidenced in Si and InGaAs photodetectors by creating defect-free geometries [117]. Recent innovations, such as femtosecond-laser-induced graphene electrodes, combine high conductivity with flexibility, facilitating advancements in wearable optoelectronics by leveraging the advantages of graphene with plasmonic effects for enhanced performance [118]. These developments address critical challenges in photodetector technology; however, scaling production and ensuring uniformity across devices remain challenges for widespread commercial adoption.

Traditional electronic and optoelectronic devices are based on commercial Si, which has a band gap between 1.1 and 1.3 eV for infrared radiation photodetection [50,54]. Another example of a photodetector fabricated by an InGaAs-based membrane photodetector plays a key role in the infrared region. However, this InGa-As-based membrane requires a complex growth process, is expensive, requires an ultralow temperature working environment, and has a specified detection wavelength, leading to trouble in practical detection measurement. Thus, it is necessary to develop well-built photodetectors with enhanced performance by introducing novel optoelectronic material, simplifying the preparation process, designing a unique structure, optimizing the preparation process, etc [119].

F.H. Alkallas et al. [120] proposed a study where a CdS/Si photodetector device was prepared by pulsed laser ablation in DMSO solution for optoelectronic applications. They studied the photodetection properties of the heterojunction of this type of device considering the influence of the laser ablation method in a liquid medium. A Cd target was ablated in a DMSO solution and stirred to facilitate the formation of CdS nano-ropes, which were subsequently deposited onto a Si-substrate via spin coating. As a II-VI group semiconductor with a direct wide bandgap of 2.42 eV, CdS is commonly utilized in transistors, sensors, and photodetectors, owing to its excellent optoelectronic properties.

**Figure 9:**
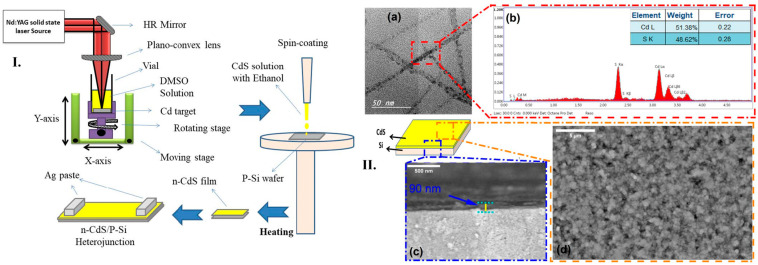
(**I**)**.** Schematic diagram of formation CdS/Si heterojunction via assisted pulsed laser ablation and spin-coating. (**II**). (**a**,**b**) TEM image and its EDX analysis of the prepared CdS nanoropes and (**c**,**d**) schematic representation and SEM image of CdS nanoropes deposited on the Si substrate from thickness and upper surface [120].

Alkallas et al. successfully synthesized CdS nanoparticles using the pulsed laser deposition (PLD) method. The process involved subjecting a cleaned Cd metal target to pulsed laser ablation (PLA) in a DMSO solution, with the target positioned at the base of a glass container. An Nd:YAG laser with a fundamental wavelength, a 10 Hz repetition rate, and a 7 ns pulse duration were used to irradiate the target with 60 mJ laser pulses. Following the reaction, the resulting CdS nanoparticle solution was centrifuged and thoroughly washed with ultra-pure water to remove residual impurities. A schematic representation of this procedure is shown in Figure 9I. To fabricate CdS/Si heterojunctions, a thin layer of CdS nanoparticles was dispersed and treated with an HF:H_2_O (1:10) solution to ensure proper adhesion. The pre-cleaned silicon wafer was then spin coated to create a uniform CdS film. Transmission electron microscopy (TEM) analysis revealed that the CdS nanoparticles had a spherical shape with an average diameter of approximately 23 nm, aligning in nano-ropes (Figure 9(IIa)). Energy-dispersive X-ray (EDX) spectroscopy confirmed the presence of Cd and S elements, while C and O were absent, indicating the high purity of CdS nanoparticles due to the efficiency of the pulsed laser ablation in liquid (PLAL) method and subsequent washing (Figure 9(IIb)). This analysis verified the successful formation of a CdS layer on the silicon substrate, ensuring good adhesion. To evaluate the influence of deposition time on film thickness and morphology, scanning electron microscopy (SEM) was performed. The SEM images showed a smooth, defect-free CdS film, with no visible cracks, pinholes, or colloidal precipitates (Figure 9(IIc)). A cross-sectional SEM image further confirmed that the CdS layer had a uniform thickness of approximately 90 nm (Figure 9(IId)). These results demonstrate that pure Cd targets ablated in DMSO solution using PLD with an Nd:YAG laser effectively produce high-quality CdS thin films. The study concluded that laser-based fabrication is a versatile method for generating micro/nanostructures with precise thickness control and defect-free morphology. Furthermore, CdS-based heterojunctions exhibited rectification behavior, indicating their strong potential for enhancing the performance of optoelectronic devices, particularly in photodetector applications. As a result, the Allkallas et al. study highlighted the excellent linearity of photodetectors produced using CdS, which significantly improves photodetection performance [120].

### 4.2. Photovoltaics

Photovoltaics is a technology that provides a practical and efficient solution to meet the growing global energy demand. It functions by converting solar energy into electrical energy, offering a sustainable and renewable alternative for power generation [50,121]. Recently, the development of photovoltaics has gained significant attention, experiencing enormous growth due to its potential for further improvement.

Solar cells are composed of semiconductor materials, where their performance and efficiency are influenced by various factors, including molecular absorption in the Earth’s atmosphere, photon energy, band gaps, and the electronic band structure governing the separation of quasi-Fermi levels. To optimize solar cell efficiency, it is crucial to consider the behavior of photons interacting with the semiconductor. Photons with energies below the band gap are not absorbed, while those with energies exceeding the band gap are not fully converted into electrical energy due to thermalization of charge carriers. Consequently, a significant portion of the incident power remains unused, particularly in semiconductors with band gaps ranging from 1.1 to 1.4 eV, where the highest spectrum-integrated power can be achieved. If a solar cell operates at a voltage corresponding to its band gap energy and a current that fully utilizes photons with energies above the band gap, it would generate maximum power output, provided that all charge carriers are efficiently collected [121]. Typically, photovoltaic solar cells use a very low amount of light due to reflection loss, but various anti-reflection structures have been designed to solve this problem [50].

In photovoltaics, laser-textured anti-reflective surfaces and back reflectors have pushed power conversion efficiencies beyond conventional limits. For instance, ultrafast laser texturing is used to create hierarchical micro/nanostructures for anti-reflective surfaces and back reflectors, improving light trapping and minimizing parasitic absorption, with textured silicon surfaces achieving over 99% light absorption across the solar spectrum [122] and reducing reflection by approximately 25% [123] or even achieving less than 3% light reflection to enhance efficiency significantly [122]. Furthermore, laser-doped selective emitters in silicon solar cells effectively reduce contact resistance while maintaining high passivation quality, which is crucial for overall device performance [123].

A novel method was proposed by H. Yang et al. [124] for processing micro/nanostructures on the surface of Cu (In, Ga)Se_2_ (CIGS)/ITO bilayer films, aiming to expand their application in the solar cell industry. CIGS thin films are widely recognized for their high efficiency in photovoltaic applications due to their direct band-gap semiconductor properties, exhibiting an absorption thickness of 1–2 µm and a high absorption coefficient of up to 10^5^ cm^−1^. Various fabrication techniques can alter the elemental composition of these films, impacting their efficiency. In this study, a femtosecond laser fabrication approach was utilized to create controllable micro/nanostructures, leveraging ultrafast laser processing to achieve unique material properties. To analyze the interaction between the CIGS/ITO bilayer films and the laser, field intensities at different layer positions were investigated, and structures were fabricated by varying laser parameters, such as scanning speed and pulse energy. The fabrication process employed an Nd:YLF femtosecond laser system, operating at an 800 nm wavelength, a 1 kHz repetition rate, and a maximum power of 5 W, delivering 120 fs pulses. The overlap of laser pulses on the sample surface was controlled using a mechanical shutter, and the CIGS/ITO films were aligned perpendicularly to the scanning direction and parallel to laser polarization. The interaction between ultrafast photons, electrons, and phonons enabled precise control over the morphology of the periodic structures by fine tuning the processing parameters during laser irradiation.

**Figure 10:**
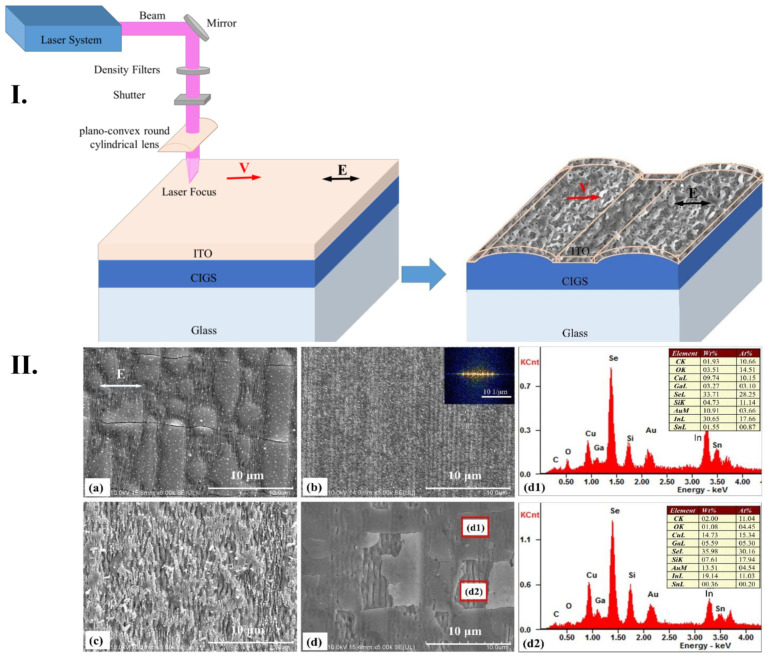
(**I**). Schematic diagram of processing: the laser irradiates the CIGS/ITO bilayer films perpendicular to their surface, and the scanning direction is parallel to the laser polarization and the fabrication of micro and nanostructures. The black arrow represents the laser polarization. (**II**). Morphology of the surface structures fabricated by using a laser pulse energy and a scanning speed of: (**a**) 0.1 µJ, 0.01 mm/s, (**b**) 0.1 µJ, 0.1 mm/s, (**c**) 0.2 µJ, 0.01 mm/s, (**d**) 0.2 µJ, 0.05 mm/s, respectively. The corresponding fast Fourier transform of (**b**) is inserted in the upper-right corner. The red rectangle in (**d**) indicates the different positions of the remaining film surface and crack, and the corresponding compositions are analyzed in (**d1**,**d2**), respectively. The white arrow represents the laser polarization [124].

Surface morphology analysis was performed using SEM and AFM. It was observed that surface structures were significantly influenced by variations in pulse energy and scanning speed. At a low pulse energy of 0.1 µJ combined with a slow scanning speed of 0.01 mm/s, the ITO thin film remained largely intact, but periodic light intensity distribution caused deformation and crack formation (Figure 10(Ia)). Increasing the scanning speed prevented deformation of the ITO layer, leading to the formation of a large modified area on the film surface (Figure 10(Ib)). At a higher pulse energy of 0.2 µJ, the top layer of the ITO film was severely damaged, while periodic surface structures were only partially retained (Figure 10(Ic)). When the scanning speed increased, fewer deformations were observed in the ITO film. Figure 10(Id) shows the bottom layer of the CIGS film, while Figure 10(Id1,d2) presents elemental composition analysis in deformed areas and cracks, indicating minimal residual ITO at the crack site.

Overall, high-spatial-frequency periodic surface structures were successfully fabricated on the ITO film when a higher spot overlap rate (scanning speed of 0.01 mm/s) was used. As the scanning speed increased and the overlap decreased, these features disappeared. By fine-tuning the pulse energy to 0.15 µJ and varying the scanning speed, ripple-like surface structures were obtained, which enhanced the optical properties and efficiency of the films. These modifications demonstrate how femtosecond laser processing can be optimized to improve photovoltaic solar cell performance, broadening their technological applications in renewable energy [124].

### 4.3. Electronic Devices

Sensors represent a key application domain where laser techniques enable unprecedented performance. Unlike optoelectronic devices, which primarily convert light to electricity (or vice versa), sensors leverage laser-induced nanostructures for mechanical, chemical, or thermal signal transduction [125]. Concretely, sensors represent a device or part of a system used to measure physical, chemical, or biological parameters, producing an equivalent signal in voltage or current form that can be measured, processed, analyzed, or transmitted. An ideal sensor is considered to be sensitive to the parameter it measures and not be influenced by other parameters from the environment or parameters that could be detected in the surroundings [126]. Sensors have an enormous impact on current industries, and several classifications have been made.

One of the most interesting and well-studied areas is the development of wearable electronics, which emphasizes user comfort, convenience, security, and improved medical functionality. They can monitor body signals, the surrounding environment, enhancing the healthcare domain. Research shows that various types of transformed sensors in wearable form can improve body signals and enable real-time sensing. Two approaches for the development of sensor system have evolved for achieving wireless functionality. One is represented by integrating wireless data or power transferable circuits into conventional sensors, and the other is based on developing sensors that detect body signals by measuring radiofrequency, both with several advantages and disadvantages. The fabrication and implementation of laser-assisted flexible sensors have become popular due to their unique advantages, including easy preparation, low production costs, and the ability to produce a wide range of raw materials. Laser-printed techniques include nanocomposite-based, laser-ablated and 3D-printed methods, and they are considered suitable for the fabrication of flexible sensors for different types of application, including wearable electronics [127].

N.A. Nikita et al. [128] proposed a special and unique method to form an electrically conductive network of multi-walled carbon nanotubes (MWCNT) in a silicone elastomer matrix to fabricate sensors sensitive to mechanical strain. Their aim is to enhance electrical conductivity and sensitivity features of the sensor using laser exposure intermediated by strong carbon nanotube networks for determining wearable electronics in the field of flexible sensors that can be attached to the human body. The development of these flexible strain sensors can detect and measure hand gestures fast and directly by placing the sensors on fingers or gloves. The fundamental principle of flexible strain sensors is the generation of several electrical signals, such as resistance, voltage, capacitance, etc. However, other types of sensors, such as piezoelectric, triboelectric or Bragg fiber array sensors, typically cannot detect slow or static strain due to rapid charge transfer. Thus, flexible strain sensors are suitable and beneficial for detecting several parameters in gesture recognition.

**Figure 11:**
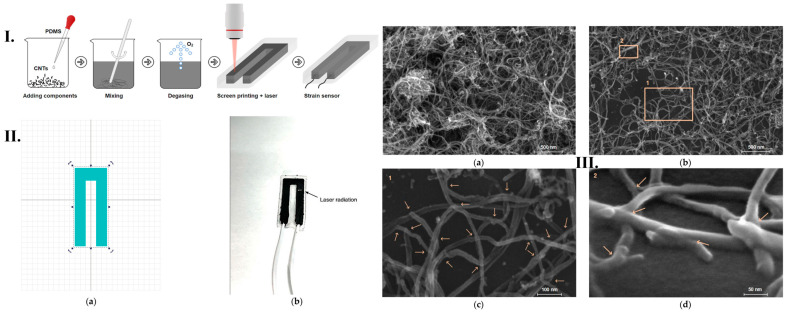
(**I**). Schematic diagram of sensor manufacturing. (**II**). Laser exposure of the active material layer of the sensor: (**a**) template for laser scanning; (**b**) active material layer of the sensor in the process of laser exposure. (**III**). Internal structure of the sensors with enlargement mode ×120,000: (**a**) fabricated without laser exposure and (**b**) with laser exposure. Enlarged areas of laser-irradiated sensors: (**c**) Area 1 with enlargement mode ×500,000, (**d**) Area 2 with enlargement mode ×1,000,000 and at 52 angle. The arrows indicate the welded areas of the nanotubes formed by the laser exposure [128].

Using laser technology to obtain the initial resistance of sensors can enhance and influence the final characteristics of sensors, such as tensile sensitivity, linearity, tensile strength or stain response. The flexible strain sensors have been manufactured consisting of electrically conductive networks of carbon nanotubes in polydimethylsiloxane (PDMS) elastomer. The surface has been scanned to form a string of electrically conductive networks of MWCNT, and the fabrication process is represented in Figure 11I. The components of PDMS elastomer have been mixed with MWCNT in equal proportions to obtain a 3 wt% MWCNT concentration, which corresponds to the percolation threshold electrical conductivity; then, the mixture was degassed in a vacuum furnace to remove air and applied to the substrate using a U-shaped screen. After the solidification of the active layer, it was exposed to laser radiation under the following parameters: 1064 nm wavelength, 0.5 J/cm^2^ energy density, 100 ns pulse duration, 30 kHz frequency and 450 mm/s beam speed. A pulsed Yb laser was used in this case, and a laser exposure pattern was programmed into the software, as shown in Figure 11II. This exposure is induced to enhance electrical performance and sensitivity due to the previously demonstrated effect of forming MWCNT networks. The electrical properties of the sensors have been compared between those obtained with and without laser exposure, using several concentrations of 2, 3 and 4 wt% of nanotubes. Figure 11III represents the structure of the electric conductive network of MWCNT sensors obtained with and without laser exposure. After laser exposure, it is observed that a redistribution of the MWCNT clusters has been performed, where large clusters are separated into smaller clusters. However, the electrical conductivity network or MWCT after laser exposure has become less complex. This type of network is suitable for strain sensors because it allows for a lower resistance hysteresis because the network stretches more uniformly and the rearrangement of the conductive network is more stable due to the absence of a large number of conductive paths. To sum up, they found that MWCNT/PDMS-based strain gauges fabricated by laser structuring of conductive networks have lower initial resistance, higher sensitivity to strain and lower hysteresis of electrical resistance compared to the structures obtained without laser exposure. Moreover, they determined a sensor-based intelligent hand gesture recognition system based on laser-formed strain sensors, with a Young’s modulus for the laser-irradiated sensor of 47 kPa, comparable to the Young’s modulus of human skin [128].

### 4.4. Light-Emitting Diode

Light-Emitting Diodes (LEDs) are fabricated from semiconductor diodes, which can emit energy when an electric current is passed through them [129]. LED represents a device that converts electrical energy into light, which is the opposite of photovoltaics. The homojunction principle of LEDs is represented by a single type of semiconductor material that forms two regions, the p-type and n-type. When these two regions are put into contact, they form a junction, and they reach thermal equilibrium after contact. The potential difference that electrons must overcome through the junction is represented by the barrier height. As the barrier is lowered due to application of a forward bias, electrons from the n-type semiconductor and holes from the p-type semiconductor are injected into the junction. Then, the electrons recombine with holes to release energy in the form of photons, which highlights the fundamental principle of the LED (AOM) [130]. The energy exhibited by these types of semiconductor diodes is in the form of photons, and the wavelength and color of the light emitted depend on the material used and on the chemical composition of the semiconductor. LEDs present several advantages, such as high-speed response time, (typically microseconds) and reduced dimensions; in comparison to conventional lighting sources, LEDs exhibit better thermal management, a long lifespan (over 50,000 h), they can be operated at various temperature ranges, among other advantages. Therefore, LEDs are regarded as superior technology capable of reducing energy consumption and reducing the emission of greenhouse gases [129]. Laser technologies are capable of producing a nano/micro-scale (LED) with enhanced electrical and optical properties (DLW).

In micro-LED manufacturing, laser lift-off (LLO) and direct laser writing (DLW) are critical technologies driving advancements in display technology. LLO enables the damage-free transfer of GaN epilayers to flexible substrates using high-energy lasers [131], enhancing the versatility of micro-LED applications. Concurrently, DLW allows for the precise fabrication of micro-LEDs smaller than 10 µm to create intricate photonic crystal arrays [132] that significantly improve light extraction efficiency by over 30% [133], contributing to improved device performance. Furthermore, recent developments in perovskite LEDs utilize laser annealing techniques to produce uniform nanocrystal arrays, achieving near-unity photoluminescence quantum yield, which is crucial for addressing scalability issues for next-generation displays [133].

**Figure 12:**
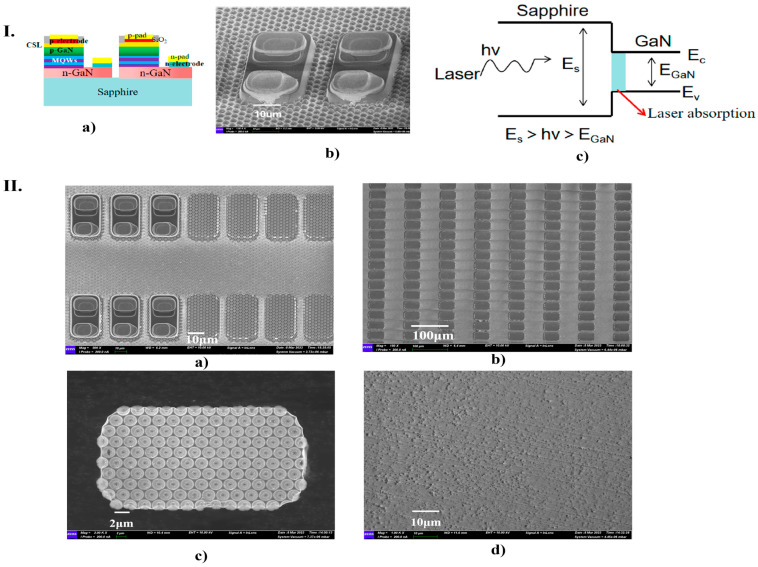
**I.** (**a**) The schematic diagram of micro-LED, (**b**) fabricated micro-LED arrays using scanning electron microscope morphology, (**c**) schematic diagram of physical mechanism of LLO process. **II.** Scanning electron microscope morphology after LLO: (**a**) the region of LLO and without LLO; (**b**) the sapphire substrate after LLO; (**c**) bottom of a single micro-LED after LLO; (**d**) the areas without micro-LED after LLO [134].

C. Liu et al. [134] proposed a study in which they analyzed optoelectrical properties of micro-LEDs before and after the laser lift-off (LLO) process. LLO has been recognized as a vital production process that facilitates the integration of micro-LEDs into display modules. They investigated this process applied to high-performance gallium nitride (GaN)-based green micro-LED arrays with a pixel size of (20 × 38) µm on a patterned sapphire substrate (PSS). This substrate has been chosen for a better epitaxial growth of GaN materials in micro-LED chips due to its cost-effectiveness and low lattice mismatch with GaN. The LLO process, using ultraviolet lasers, is used because the photon energy of the light source overcomes the bandgap of GaN but remains below that of sapphire. The selective absorption of laser energy by the GaN layer causes a rapid increase in temperature at the interface, which exceeds the thermal delamination temperature, leading to the thermal decomposition of GaN into nitrogen gas (N2) and low-melting-point gallium metal. After the applied process, micro-LEDs highlighted significant enhancements in light output power and external quantum efficiency. In order to obtain enhanced performance of Micro LEDs, they used a 4-inch PPS substrate where the micro-LED array structure is formed of multiple layers from bottom to top: sapphire substrate layer, GaN buffer layer, N-type layer, GaN layer, active layer with several quantum wells, P-type GaN contact layer, current spreading layer and p-type electrode. Each micro-LED had a 20 µm × 38 µm with a pixel horizontal distance of 6 µm and vertical distance between 50 µm between these two chips. Figure 12I presents the schematic diagram of micro-LED arrays and the morphology of micro-LED arrays as observed using a SEM. LLO is performed to remove the sapphire substrate by using a short-wavelength laser with photon energy greater than GaN bandgap but smaller than sapphire with the laser strongly absorbed by sapphire. In their experiment, the photon energy of the semiconductor laser was 4.83 eV, falling between the bandgaps of the sapphire substrate (Es) and the GaN (EGaN), as shown in Figure 12(Ic). After the LLO process, no chip fragmentation or chips falling off have been observed. The LLO parameters were the following: 257 nm, power 0.8 W, fill density 13 µm, pitch 13 µm, frequency 200 kHz and scan speed 2600 mm/s. This process has been validated throughout SEM images (Figure 12II). It is observed that in a region without LLO, the boundary is clearly defined as the separation was completed (Figure 12(IIa)). The toughness of the LLO process and the successful transfer are represented in Figure 12(IIb), while the smoothness and integrity of the PSS interface are demonstrated in Figure 12(IIc). Thus, no visible damage has been observed between the GaN film and the sapphire substrate, indicating the successful removal of GaN film after LLO (Figure 12(IId)). Therefore, micro-LEDs remained intact after removing the PSS during LLO. Notable improvements have been demonstrated, including enhanced light extraction and heat dissipation, with the full width at half maximum (FWHM) reduced by approximately 10%. Additionally, the LLO process has had a minimal effect on the electrical characteristics of the micro-LEDs. In this way, they proved a significant enhancement of optoelectronic devices, such as LEDs using laser techniques [134].

### 4.5. Quantum Optoelectronics

Laser-fabricated nanostructures are pivotal for creating advanced quantum light sources with precisely controlled emission characteristics. For instance, femtosecond laser annealing of InAs/GaAs has been demonstrated to create site-controlled quantum dot arrays with an impressive 85% spatial uniformity, paving the way for scalable quantum photonic circuits [135]. Furthermore, gold bowtie plasmonic nanocavities fabricated using two-photon polymerization have shown a remarkable 50-fold enhancement in single-photon emission rates through the Purcell effect, a crucial advancement for quantum communication technologies [136]. Additionally, the integration of these sources with micro optocoupler technology, such as detecting radiation from an InGaAs/GaAs quantum well dot (QWD) microdisk laser using a nearby photodiode fabricated from the same epitaxial heterostructure, showcases the potential for on-chip quantum photonic integration [137].

### 4.6. Flexible Optoelectronics

Laser-induced graphene (LIG) patterning on polyimide substrates enables the fabrication of advanced flexible optoelectronic devices. For instance, LIG-based foldable photodetectors have demonstrated excellent mechanical stability, retaining 90% of their responsivity even after 10,000 bending cycles [138]. Furthermore, LIG electrodes integrated into stretchable perovskite LEDs (PeLEDs) have shown superior performance compared to conventional ITO electrodes, maintaining 15% external quantum efficiency (EQE) at a significant strain of 50% [139].

### 4.7. Supercapacitors

Laser-fabricated techniques offer high resolution for the design and fabrication of miniaturized supercapacitors. One prominent approach involves the laser reduction of graphene oxide films, where a high-power laser beam selectively patterns and reduces the graphene oxide to create conductive electrodes. Another method directly utilizes laser-enabled graphene as the electrode material; the superior conductivity, chemical stability, and high surface area of laser-induced graphene facilitate the formation of efficient electric double-layer capacitors [140].

## 5. Conclusions

Micro/nanostructures fabricated by different types of laser processing provide a pivotal manufacturing technique for enhancing the performance of optoelectronic devices. The key features of laser fabrication are the precise control of morphology, high-speed deposition or removal of material, and a high-quality approach. Techniques such as laser ablation, LIPSS, TPP, or laser exposure offer various advantages, including fast processing, cost effectiveness, and a high degree of design freedom, providing a facile approach to manufacturing customizable micro/nanostructures. Also, it avoids using toxic compounds or hazardous reactants, being environmentally friendly. The flexibility of laser techniques, which have found their application in various domains and approaches, such as remelting, evaporation, or even thin-coating formation, allows the suitable production of micro/nanostructures without the necessity of complex methods, such as lithography. This enhances the versatility of device designs, especially in optoelectronic devices, contributing to the development of semiconductors used in photodetectors, photovoltaics, sensors, biosensors or LEDs, improving their efficiency and performance. In conclusion, laser-fabricated micro/nanostructures have become an indispensable technique for advancing optoelectronic devices, contributing to an ideal solution for improving their performance. Many research articles and reviews cover and analyze laser-fabricated structures. However, further studies and analysis are still required to investigate significant features such as the influence of the parameters over the material, unexplored regions, or the relation between the laser and the final properties of the structures. Future experiments should cover laser technologies as a critical aspect of the development of future novel devices, overcoming current challenges in a wider range of applications such as communications, biomedical, energy, optical, and many more.

## 6. Future Perspectives and Challenges

The field of laser-fabricated micro/nanostructures for optoelectronic devices has seen remarkable advancements over the past few decades, with significant improvements in device performance, fabrication precision, and material versatility. However, as the demand for more efficient, compact, and multifunctional optoelectronic devices continues to grow, several challenges and opportunities for future research emerge. This section outlines key areas that require further exploration and innovation to fully realize the potential of laser-based fabrication techniques in optoelectronics.

### 6.1. Scalability and Industrial Integration

The scalability of laser-fabricated micro/nanostructures for industrial production presents significant challenges despite the high precision and flexibility offered by laser processing. Future advancements must focus on developing cost-effective, high-throughput laser systems that can replicate the precision of laboratory setups. Additionally, integrating laser fabrication with existing semiconductor manufacturing processes could lead to innovative hybrid techniques. While ultrafast laser techniques can achieve resolutions below 100 nm, translating this capability to large-scale production remains a hurdle [141].

The scanning laser interference method has shown promise in producing micro/nanostructures at high speeds and low costs, indicating potential pathways for scalability [142].

Combining laser fabrication with traditional methods, such as photolithography, could enhance production capabilities, allowing for the creation of complex structures with high design flexibility [143].

Laser-assisted methods have been effective in developing nanostructures for electronic devices, suggesting a strong synergy with existing semiconductor processes [144].

The industrial adoption of laser-based micro/nanofabrication faces significant challenges in throughput, where current Laser-Induced Periodic Surface Structures systems achieve approximately 1 cm^2^/min, falling short of the >100 cm^2^/min requirement for semiconductor fabrication, suggesting that multiplexed beam architectures and high-repetition-rate lasers (>10 MHz) offer potential solutions. Integration into existing semiconductor workflows, such as hybrid laser-photolithography approaches using laser-defined alignment marks combined with extreme ultraviolet patterning, has shown promise in reducing costs by up to 30% compared to standalone laser systems [145]. Finally, the high cost of femtosecond lasers remains a barrier, although advancements in fiber laser technology project a potential fivefold reduction in prices within the next five years [146].

Conversely, while laser techniques show promise, traditional methods, such as photolithography and chemical vapor deposition, remain dominant in semiconductor manufacturing, highlighting the need for further research to bridge these technologies effectively.

### 6.2. Material Compatibility and Novel Materials

Lasers have proven effective in fabricating micro/nanostructures across various materials, yet their interaction with emerging materials, such as two-dimensional (2D) materials, remains underexplored. The unique properties of 2D materials, such as graphene and transition metal dichalcogenides (TMDs), present opportunities for advancements in optoelectronic devices. However, understanding the effects of laser parameters on these materials is crucial for optimizing their performance.

Laser-assisted methods have been shown to effectively synthesize graphene, enhancing its properties for applications in electronics [144].

The interaction of lasers with TMDs is still being investigated, with challenges in nanoscale synthesis noted [147].

Laser techniques have also shown significant promise in patterning 2D materials and perovskites, which are pivotal for next-generation optoelectronic applications. For 2D materials, such as graphene and transition metal dichalcogenides (TMDs), ultrafast laser processing enables precise etching and doping without introducing excessive thermal damage, preserving their intrinsic electronic and optical properties [148]. For example, femtosecond lasers can create nanoscale pores in graphene, enhancing its plasmonic properties for sensor applications. In the case of perovskites, laser-assisted methods allow for controlled crystallization and patterning, which are critical for achieving high-performance solar cells and LEDs. The localized energy deposition of lasers minimizes thermal degradation, enabling the fabrication of perovskite microarrays with uniform optoelectronic properties [149].

Furthermore, the incorporation of 2D materials into halide perovskite optoelectronic devices (HPODs) provided a significant enhancement. These types of devices can be used as charge transport media, encapsulation layers for photodetectors, benefiting from their special and unique structures and properties. HPODs could provide a bright future in the domain of 2D materials and perovskites, along with the fast advancement and understanding of this domain [150]. These advancements underscore the versatility of laser techniques in addressing the challenges of patterning emerging materials.

Variations in laser fluence can significantly affect the structural and electronic properties of materials, as demonstrated in studies on graphene oxide and silicon composites [151].

The nanoscale effects of laser processing on materials, such as perovskites, are not yet fully understood, indicating a need for further research [152].

While the potential of lasers in processing 2D materials is promising, the complexities of their interactions at the nanoscale necessitate comprehensive studies to fully harness their capabilities in optoelectronic applications.

### 6.3. Advanced Laser Techniques and Multifunctional Structures

The advancement of laser techniques, such as multi-photon lithography, ultrafast laser processing, and laser-induced forward transfer, is revolutionizing the fabrication of multifunctional micro/nanostructures. These methods facilitate the creation of complex 3D architectures and hybrid materials, enabling devices with integrated functionalities, such as sensing, energy harvesting, and light emission [153].

For example, laser direct writing (LDW) offers a promising alternative to traditional lithography, particularly in semiconductor fabrication. This technique allows for the precise control of micro/nano-scale structures, essential for advanced applications in microelectronics and photonics [154].

Another method, laser-induced forward transfer, is a sustainable and precise method for printing metallic patterns and solder materials, crucial for photonic applications. It allows for the reproducible printing of electrodes, facilitating the development of all-printed electronic devices [155,156].

While these advanced laser techniques present significant opportunities for innovation in optoelectronic devices, challenges remain in optimizing parameters for specific applications and ensuring scalability in industrial settings. Future research should address these challenges to fully realize these technologies’ potential.

### 6.4. Integration with Emerging Technologies

The integration of laser-fabricated micro/nanostructures with emerging technologies, such as flexible electronics and the Internet of Things (IoT), offers significant research opportunities. Laser techniques enable the precise fabrication of micro/nanostructures on unconventional substrates, which is essential for developing flexible and stretchable optoelectronic devices. LDW allows for the regulation of material properties, enabling the creation of complex structures on flexible substrates, such as polymers and textiles [157,158].

Laser-based methods facilitate the production of soft electronic components, including sensors and actuators, which are crucial for wearable devices and healthcare monitoring [158].

Future studies should focus on combining laser-fabricated structures with wearable technologies to enhance functionalities, such as real-time health monitoring and energy harvesting [159,160].

The potential for adaptive optical systems using laser-fabricated micro/nanostructures could revolutionize various applications in smart devices and IoT [157].

While the advantages of laser fabrication are clear, challenges remain in scaling these technologies for mass production and ensuring compatibility with diverse materials. Further exploration is needed to address these issues and fully realize the potential of these innovations.

### 6.5. Challenges in Characterization and Modeling

As the complexity of laser-fabricated micro/nanostructures increases, advanced characterization techniques and predictive modeling become essential. Understanding the interplay between laser parameters, material properties, and device performance is vital for optimizing fabrication processes. Future research should prioritize in-situ characterization methods for real-time monitoring of laser–material interactions, alongside computational modeling and machine learning to predict fabrication outcomes.

In-situ characterization can provide real-time insights into laser–material interactions, enhancing understanding of the fabrication process [161].

Standardized material characterization procedures are necessary to accurately determine parameters, such as absorption coefficients and ablation thresholds, which are critical for effective laser processing [162].

Computational models can simulate laser ablation processes, predicting outcomes, such as ablation shape and temperature distribution, thus reducing development time [162].

Machine learning can be integrated to optimize laser parameters, improving the design of structures with tailored properties [144].

While the focus on advanced techniques is crucial, it is also important to consider the limitations of current methods, such as the need for extensive experimental validation of predictive models to ensure reliability in practical applications. In conclusion, while laser-fabricated micro/nanostructures have already made significant contributions to the field of optoelectronics, there is still ample room for innovation and improvement. Addressing the challenges outlined above will advance state-of-the-art laser fabrication and open up new possibilities for the design and application of next-generation optoelectronic devices. By leveraging the unique capabilities of laser technology, researchers can continue to push the boundaries of what is possible in optoelectronics, paving the way for a future of smarter, more efficient, and more sustainable devices.

## Figures and Tables

**Table 1:** Key aspects of laser techniques used for micro/nanostructures fabrication.

Laser Technique	Key Features	Advantages	Applications	References
Laser-induced periodic surface structures (LIPSS)	Utilizes femtosecond lasers to create periodic subwavelength structures. Influenced by factors, such as fluence, wavelength, pulse number, material properties, and polarization.	Low sensitivity to material type, high stability, processing efficiency, and enhanced wetting properties.	Surface functionalization, photocatalysis, and hierarchical structuring.	[78]
Two-photon polymerization (TPP)	A femtosecond laser-driven photochemical process that enables high-resolution 3D micro/nanofabrication in photosensitive resins.	High spatial resolution, 3D fabrication capability, and biocompatibility.	Biomedical scaffolds, photonics, microfluidics, and bioelectronics.	[79]
Laser ablation	Direct removal of material from solid surfaces using pulsed lasers. Generates high-energy interactions leading to localized heating and vaporization	High precision, cost effectiveness, and suitable for a wide range of materials.	Nanoparticle synthesis, thin-film deposition, and micro patterning.	[80]
Hybrid laser processing	Combines multiple laser techniques to fabricate complex micro/nanostructures with tailored properties.	Enables multifunctional structuring, enhanced surface properties, and scalability.	Biomedical implants, functional coatings, and energy storage devices.	[81]

**Table 2:** Performance benchmarks of laser techniques. EQE: External Quantum Efficiency.

Technique	Resolution (nm)	Speed (mm^2^/s)	Material Range	EQE Improvement	Reference
LIPSS	50–500	0.1–10	Metals, Semiconductors	15–30% (photodetectors)	[86]
TPP	10–200	0.001–0.1	Polymers, Resins	20–40% (LED light extraction)	[87]
Femtosecond Ablation	20–100	0.01–1	Broad (incl. 2D materials)	10–25% (PV antireflection)	[88]

## Data Availability

No new data were created or analyzed in this study. Data sharing is not applicable to this article.
